# Lgr6^+^ cells in the biological system during homeostasis and injury

**DOI:** 10.1016/j.gendis.2025.101863

**Published:** 2025-09-24

**Authors:** Han Li, Xiaoqi Guan, Yu Wang, Haidong Guo

**Affiliations:** a Academy of Integrative Medicine, Shanghai University of Traditional Chinese Medicine, Shanghai 201203, China; b Department of Neurology, Shuguang Hospital Affiliated to Shanghai University of Traditional Chinese Medicine, Shanghai 201203, China

**Keywords:** Biomarker, Lgr6, Progenitor cells, Signalingpathways, Tissue injury

## Abstract

Lgr6 has attracted significant attention in biomedical research in recent years. As a member of the G protein-coupled receptor family, Lgr6 plays a crucial role in the occurrence and development of various diseases, including diabetic cardiomyopathy, bone regeneration defects, and skin injury repair, where it is vitally involved in cellular signal transduction. This study endeavors to investigate the distribution and functions of Lgr6^+^ cells across organisms, particularly during homeostasis and damage scenarios. Lgr6^+^ expression occurs across skin, mammary glands, kidneys, and intestines, crucial for development and tissue repair. Abnormal expression of Lgr6 is also observed in the onset and progression of major systemic diseases, especially in tumors. Thus, Lgr6 has been identified as a promising therapeutic target for cancer and other diseases, influencing their onset, progression, and treatment.

## Introduction

The leucine-rich repeat-containing G protein-coupled receptor 6 (Lgr6) has really taken center stage in biomedical research lately. The Lgr family forms a subset of G-protein-coupled receptors that are particularly rich in leucine-rich repeat sequences. This group is pivotal in the realms of stem cell biology, tissue repair, and cancer. Within this family, Lgr4 is the standout member, actively managing multi-organ development and the maintenance and regeneration of adult tissues, all through the central process of R-spondin (RSPO)-wingless/integrated (Wnt) signal escalation. Lgr5, on the other hand, is a pivotal molecule in the Wnt signaling cascade and a signature receptor on adult stem cells. Its presence is precisely confined to the bustling hubs of active stem cells in tissues with high turnover rates, such as the base of the crypt columnar stem cells, the foundation of gastric gland stem cells, the robust hair bulge multipotent stem cells, and the bile duct/ductal progenitor cells. Being part of the G protein-coupled receptor (GPCR) family, Lgr6 is a real jack-of-all-trades in organisms, influencing everything from cell growth and specialization to programmed cell death.^1^ A distinguished feature of Lgr6 is within its extracellular domain, which contains multiple leucine-rich repeat sequences. These sequences are crucial for protein–protein interactions.

**Figure 1:**
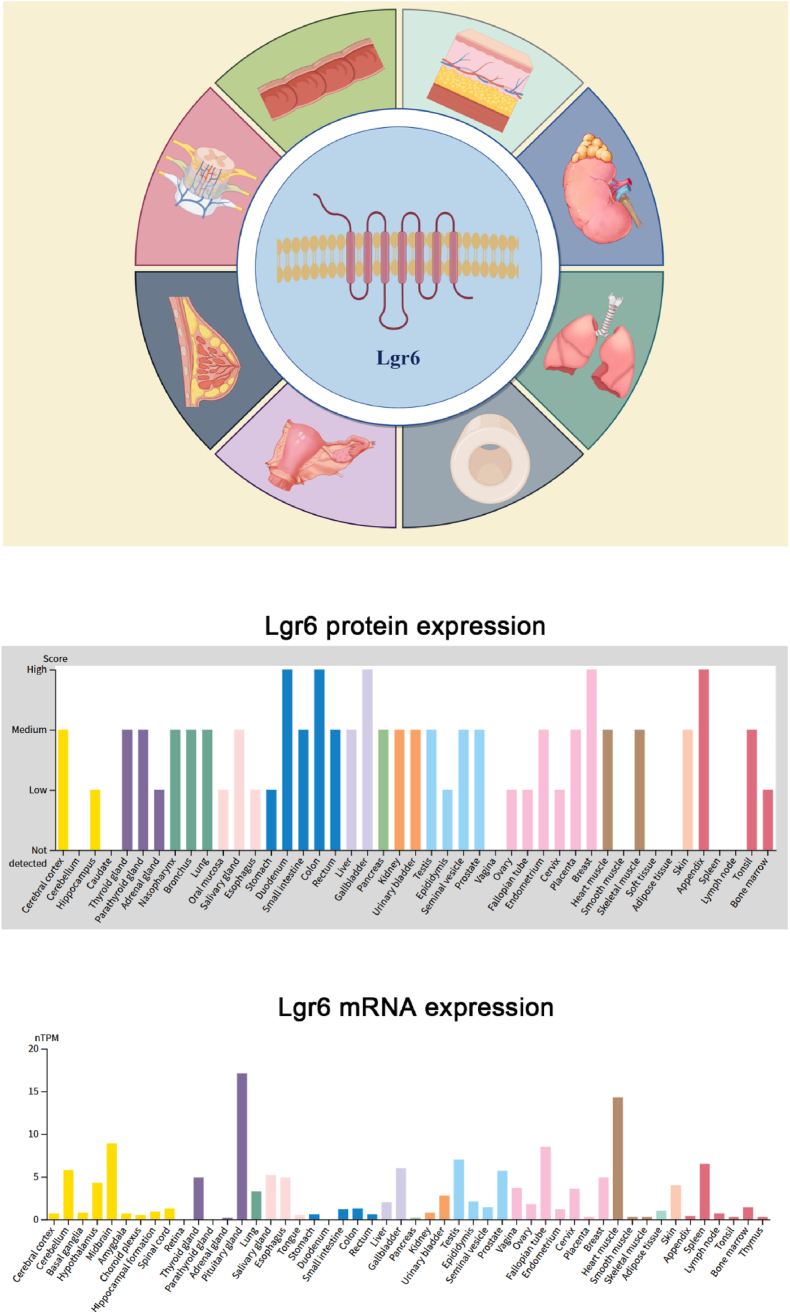
Expression of Lgr6 in different organs.

Lgr6 expression occurs in diverse tissues, notably skin,^2^, ^3^, ^4^ mammary gland,^2^^,^^5^ kidney,^5^ and intestine (including its accessory structures),^6^ and plays key roles in embryonic development, including regulation of neural tube closure, limb development, and organ formation (Fig. 1). Moreover, Lgr6 plays a role in adult tissue regeneration and repair, including skin wound healing,^7^ growth, and development.^2^ The Lgr6 receptor also significantly influences the onset and progression of certain diseases. For example, Lgr6 is expressed abnormally in various cancers like breast cancer,^4^ which may promote tumor initiation, progression, and metastasis. Additionally, Lgr6 is linked to rectal cancer^8^ and renal disorders.^9^, ^10^, ^11^, ^12^, ^13^ In the kidney, Lgr6 regulates the proliferation and differentiation of renal tubular epithelial cells.^2^^,^^14^

Given Lgr6's critical function in diverse physiological and pathological pathways (Table 1), it represents a potential therapeutic target. For example, targeting the expression or activity of Lgr6 may be beneficial to understanding the development of physiopathology in various systems, such as the urinary, respiratory, skeletal, reproductive, nervous, and digestive systems. In addition, due to the specific expression of LGR6 in some cancers, it is also considered a promising target for cancer treatment (Table 2).

**Table 1:** The effects of Lgr6^+^ cells on different tissues under homeostasis and injury conditions.

System	Tissue context	Expression status	Functional outcome	Clinical relevance
Urinary system	Kidney development/adult kidney	↑ in nephron progenitor cells (E11.5)^14^	Promotes nephrogenesis via mesenchymal-to-epithelial transition^2^^,^^14^	/
	Diabetic kidney disease	↓ in diabetic kidney disease^9^^,^^10^	Reduces ROS/fibrosis via cAMP-SOD2^22^	MaR1/Lgr6 axis → antioxidant/anti-inflammatory
				Markers of diabetic kidney disease severity
Respiratory system	Normal lung tissue	↑ Stem cell repair activity^29^	Scgb1a1^+^ cells → airway epithelial cells	/
			Lgr6^+^ stem cells → secrete SDF-1^27^^,^^29^	
	Non-small cell lung cancer	Lgr6^+^ cells > Lgr6^–^ cells (tumorigenic ability)^21^	Enhances tumorigenicity & stemness^21^	High Lgr6 = poor prognosis (stage III-IV)^21^
	Chronic obstructive pulmonary disease/idiopathic pulmonary fibrosis	↑ in fibrotic regions^30^^,^^39^	Drives Wnt hyperactivation → senescence^30^	Biomarker for disease progression^39^
Skeletal system	Physiological bone formation/fracture healing	Osteogenic differentiation of mesenchymal stem cells→↑ early differentiation/↓ differentiation and maturation^43^, ^44^, ^45^/Lgr6↑ → repair ability↓^47^	Wnt/β-catenin → β-catenin → ↑osteogenic differentiation and mineralization^48^^,^^50^/Synergistically regulate bone formation (BMP)^53^, ^54^, ^55^	/
	Intervertebral disc degeneration	Lgr6↓ (can be reversed by MaR1)^57^	↑MERTK/AXL/CX3CR1; ↑COL2A1; ↓MMP13; ↑BCL2, ↓caspase-3/BAX	MaR1-Lgr6 agonists (such as pioglitazone to enhance phagocytic function)^57^
Genital system	Cervical cancer	Lgr6↑ → CSCs/SOX2^+^/OCT4^+^^63^	↑Wnt/β-catenin → ↑SOX2/OCT4 → ↑self-renewal and tumorigenicity^63^; the TCF7L2-β-catenin complex drives Lgr6 transcription^63^^,^^65^	Blocking the Lgr6-TCF7L2 circuit may inhibit CSCs^63^
		TCF7L2 + Lgr6 → positive feedback^63^^,^^65^		
	Ovarian cancer	Lgr6↑^59^^,^^62^	Lgr6^+^ R-spondin → maintenance and transfer of CSCs dryness^15^^,^^61^; ↓Lgr6 → reverse chemotherapy resistance^62^	Poor chemotherapy response and low survival rate^60^
	Breast cancer	↑ in luminal progenitors	Drives tumor initiation^5^	ER-negative subtype biomarker^66^
Nervous system	Cortical astrocytes	↑ in layer 5 (8.3-subset)^69^^,^^70^^,^^72^; regulated by neuron-derived RSPO1^68^^,^^71^	Wnt signaling pathway mediation; Norrin + Lgr6 → autocrine/paracrine circuit; ↓ IL-1β and IL-6; ↑IL-10^69^^,^^70^	Lgr6/Norrin signal↓→Synaptic loss (Alzheimer), abnormal dendritic spines (Autism)^69^^,^^70^; MaR1 (Lgr6 agonist) → ↓Neuroinflammation after subarachnoid hemorrhage
Digestive system	Colorectal cancer	↑ in colorectal cancer organization	↑PI3K/AKT → ↑proliferation and inhibit apoptosis^73^; ↑Wnt/β-catenin signaling → ↑Myc/β-catenin → drive transfer^15^; ↓Lgr6 → ↓MMP9/β-catenin → Prevent invasion^84^	High expression indicates advanced TNM (stage Ⅲ/Ⅳ), lymph node metastasis, and shortened disease-free survival^81^
	Gastric cancer	↑ in advanced gastric cancer (stage Ⅲ/Ⅳ) and lymph node metastasis^79^^,^^83^; the expression of Lgr6 and Twist1 is positively correlated.^85^^,^^87^	↑PI3K/AKT/mTOR pathway → ↑tumor growth^83^; Lgr6/Twist1 axis → E-Cad↓/vimentin↑^87^; regulating MMP9 → enhancing matrix degradation capacity^84^	The expression level is positively correlated with the depth of invasion and lymph node metastasis^79^^,^^83^; ↓Lgr6 → ↓the proliferation and invasion of gastric cancer cells^84^
	Pancreatic ductal adenocarcinoma	Wnt activation →Lgr6↑→ Further amplify the Wnt signal	Promotes Twist1-mediated epithelial-mesenchymal transition^87^	Wnt inhibitors + Lgr6-siRNA → block the feedback loop (preclinical model)^87^
Cardiovascular system	Hypertensive remodeling	LGR6 expression loss → serum MaR1 level ↓	LGR6 deficiency → blood pressure ↑, thickening/fibrosis of the vascular media, phenotypic transformation of vascular smooth muscle cells ↑, pyroptosis ↑	The vascular protective effect of MaR1 depends on LGR6; LGR6 is a potential GPCR target for the treatment of hypertension^11^^,^^19^^,^^20^^,^^88^
	Pulmonary arterial hypertension (PAH)	Serum MaR1↓ → in patients with Lgr6↓ PAH^89^; monocrotaline/hypoxia model lung tissue Lgr6↑^89^	MaR1 improves PAH through LGR6^11^^,^^89^	The role of LGR6 as a MaR1 receptor in PAH needs further verification
				Different models (SuHx *vs*. monocrotaline/hypoxia) may affect the function of LGR6^11^^,^^42^^,^^89^
	Myocardial ischemia-reperfusion injury	LGR6↓ (Myocardial Tissue)	LGR6 KO → Infarct area↑, myocardial enzymes↑, necrotic apoptosis↑	RSPO3-LGR6-STAT2-ZBP1 axis (potential therapeutic target)^90^, ^91^, ^92^, ^93^, ^94^, ^95^, ^96^, ^97^, ^98^, ^99^
			LGR6 overexpression → reduces damage	
	Diabetic cardiomyopathy	Lgr6↑	LGR6 KO → Cardiac function ↓, myocardial hypertrophy/fibrosis ↑; LGR6 overexpression → improved phenotype	LGR6-STAT3-Pgc1α signaling axis (potential therapeutic target)^112^^,^^113^
	Myocardial hypertrophy due to pressure overload	LGR6↓	Lgr6 knockdown → Myocardial hypertrophy/dysfunction/metabolic remodeling ↑	Lgr6-USP4-PPARα signaling axis (potential therapeutic target)^112^, ^113^, ^114^, ^115^^,^^132^
			Lgr6 overexpression → improved phenotype

**Table 2:** Abbreviation.

Abbreviation	Full name	Contextual description/definition
AKT	Protein kinase B	Serine/threonine kinase in the PI3K/AKT pathway
ALP	Alkaline phosphatase	Enzyme marker for osteoblast activity and bone formation
AngII	Angiotensin II	Peptide hormone inducing hypertension; used in vascular remodeling models
ANP/BNP	Atrial/B-type natriuretic peptide	Biomarkers of myocardial hypertrophy and heart failure
AQP5	Aquaporin 5	Water channel protein implicated in tumor invasion
AMP	Adenosine monophosphate	It is an ester of phosphoric acid and ribonucleotide, and is composed of phosphate functional groups, pentose nucleic sugars, and the base adenine
BMP	Bone morphogenetic protein	Growth factors inducing bone and cartilage formation
BMSCs	Bone marrow stromal cells	Mesenchymal stem cells derived from bone marrow
CaMKII	Calcium/calmodulin-dependent kinase II	Enzyme regulating vascular smooth muscle cell contraction; inhibited by MaR1-LGR6 in hypertension
cAMP	Cyclic adenosine monophosphate	Second messenger in GPCR signaling, regulates metabolism/gene expression
CM cells	Cap mesenchyme cells	Progenitor cells in embryonic kidney development
COPD	Chronic obstructive pulmonary disease	Progressive lung disease characterized by airflow obstruction
CRC	Colorectal cancer	Cancer of the colon or rectum
DKD	Diabetic kidney disease	Kidney damage resulting from chronic diabetes
EMT	Epithelial-mesenchymal transition	Process where epithelial cells lose adhesion and gain migratory/invasive properties
EPAC1	Exchange protein activated by cAMP 1	Mediates cAMP signaling in LGR6-dependent cardioprotection
ERK	Extracellular signal-regulated kinase	Kinase in MAPK pathway; phosphorylated by MaR1-LGR6
ESCC	Esophageal squamous cell carcinoma	Aggressive cancer subtype; LGR6 overexpression correlates with poor prognosis
ETC	Electron transport chain	Mitochondrial complex; upregulated by LGR6-STAT3-Pgc1α axis in diabetic cardiomyopathy
GPCR(s)	G protein-coupled receptor(s)	Cell surface receptors that transduce extracellular signals via G proteins
HG	High glucose	*In vitro* model for diabetic injury
HNSCC	Head and neck squamous cell carcinoma	SCC occurring in the oral cavity, pharynx, or larynx
HPV	Human papillomavirus	Virus linked to cervical, oral, and other cancers
I/R	Ischemia-reperfusion	Injury model
IFE	Interfollicular epidermis	Skin compartment maintained by LGR6⁺ stem cells
IPF	Idiopathic pulmonary fibrosis	Chronic lung disease involving irreversible scarring of lung tissue
IVDD	Intervertebral disc degeneration	Degeneration of spinal discs, leading to back pain and reduced mobility
Lgr6	Leucine-rich repeat-containing G protein-coupled receptor 6	A receptor in the GPCR family, key in stem cell regulation, tissue repair, and disease progression
LRP6	Low-density lipoprotein receptor-related protein 6	Coreceptor in the Wnt signaling pathway
LRR(s)	Leucine-rich repeat(s)	Structural motifs in proteins involved in ligand binding/protein interactions
LSCs	Lung stem cells	LGR6⁺ progenitors maintaining lung homeostasis
MAPK	Mitogen-activated protein kinase	Enzyme family involved in cellular stress responses
MaR1	Maresin 1	Pro-resolving lipid mediator with anti-inflammatory and regenerative effects
MaSCs	Mammary stem cells	Mammary stem cells that reside in the mammary tissue and have the capacity for self-renewal and differentiation into all mammary epithelial cell types
MET	Mesenchymal-to-epithelial transition	Cellular process where mesenchymal cells acquire epithelial properties
MSCs	Mesenchymal stem cells	Multipotent stromal cells capable of differentiating into bone, cartilage, or fat
mTOR	Mammalian target of rapamycin	Kinase regulating cell proliferation and metabolism
NLRP3	NLR family pyrin domain containing 3	Inflammasome component; promotes pyroptosis in hypertension when LGR6 is deficient
NPCs	Nephron progenitor cells	Embryonic kidney cells that differentiate into nephrons
NSCLC	Non-small cell lung cancer	Most common type of lung cancer
OA	Osteoarthritis	Degenerative joint disease involving cartilage loss
OCN	Osteocalcin	Protein produced by osteoblasts, involved in bone mineralization
OPN	Osteopontin	Synthetic marker for vascular smooth muscle cell phenotypic switching; upregulated in hypertension
PAAT/PET	Pulmonary artery acceleration time/Pulmonary ejection time	Right heart function parameters improved by MaR1-LGR6 in PAH
PAH	Pulmonary arterial hypertension	Vascular disorder; LGR6 roles vary by model
PASMC	Pulmonary artery smooth muscle cell	Key cell type in PAH pathogenesis; proliferation inhibited by MaR1-LGR6
PDAC	Pancreatic ductal adenocarcinoma	Aggressive cancer; LGR6 amplifies Wnt-Twist1-EMT axis
PI3K	Phosphatidylinositol 3-kinase	Enzyme in the PI3K/AKT pathway, regulating cell growth/survival
PPARα	Peroxisome proliferator-activated receptor α	A member of the nuclear-receptor superfamily of ligand-dependent transcription factors related to retinoid, steroid, and thyroid hormone receptors
ROS	Reactive oxygen species	Chemically reactive molecules causing oxidative stress and cellular damage
RSPO1	R-spondin 1	Ligand that amplifies Wnt signaling by binding to LGR receptors
RSV	Respiratory syncytial virus	Common virus causing respiratory infections
SCC	Squamous cell carcinoma	Cancer arising from squamous epithelial cells (*e.g.*, skin, lung, esophagus)
SDF-1	Stromal cell-derived factor 1	Chemokine secreted by LGR6⁺ lung stem cells to promote repair
SOD2	Superoxide dismutase 2	Mitochondrial enzyme that neutralizes ROS
Tregs	Regulatory T cells	Immune cells that suppress excessive immune responses
STAT2/3	Signal transducer and activator of transcription 2/3	Transcription factors; STAT2 drives necroptosis, STAT3 represses Pgc1α in dilated cardiomyopathy
SuHx	Sugen 5416 + hypoxia	Common PAH mouse model; LGR6 downregulated here
TCF7L2	Transcription factor 7-like 2	Wnt pathway transcription factor; forms feedback loop with LGR6 in cervical cancer
TNFα	Tumor necrosis factor alpha	Pro-inflammatory cytokine; triggers fibroblast activation in lung repair
Tregs	Regulatory T cells	Immune cells suppressing excessive responses; modulated by LGR6-MaR1 in RSV
USP4	Ubiquitin-specific protease 4	Deubiquitinase stabilizing PPARα; activated by LGR6 in cardiac hypertrophy
VSMC	Vascular smooth muscle cell	Key cell type in vascular remodeling; phenotype switching regulated by LGR6-MaR1
Wnt	Wingless/Integrated	Signaling pathway critical for stem cell maintenance and development
ZBP1	Z-DNA binding protein 1	Necroptosis driver; activated by STAT2 in LGR6-deficient hearts

## The structure of Lgr6

**Figure 2:**
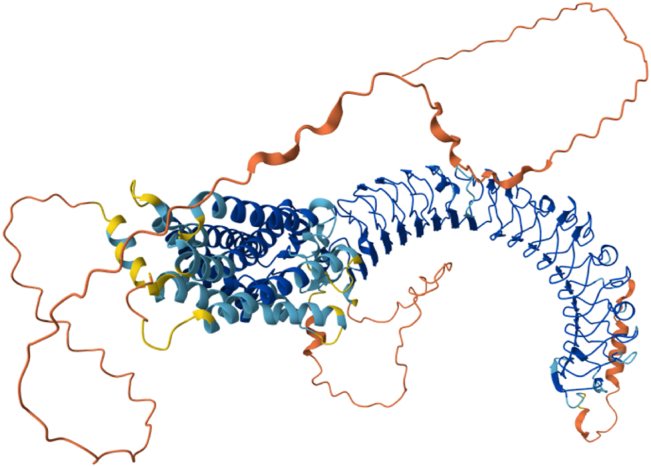
The structure of Lgr6.

The Lgr6 receptor is composed of three primary components: the N-terminal, transmembrane region, and C-terminal (Fig. 2). The N-terminal encompasses a signal peptide that directs the protein into the endoplasmic reticulum for post-translational modification. This region is abundant in leucine-rich repeats (LRRs), which form a horseshoe structure capable of binding with various ligands, thereby activating or inhibiting downstream signaling pathways. The transmembrane region consists of seven transmembrane helices that anchor the receptor to the cell membrane. The C-terminal contains a leucine-rich repeat, a characteristic feature of the LRR family. Lgr6 transmits signals by coupling with G proteins. Upon ligand binding to receptor, the α subunit of the G protein dissociates from the βγ subunit, thereby activating downstream signaling pathways. These pathways, encompassing phosphatidylinositol 3-kinase (PI3K), protein kinase B (PKB/Akt), and mitogen-activated protein kinase (MAPK), are instrumental in governing essential cellular functions. Ultimately, they exert control over processes like cell growth, differentiation, and apoptosis.

Lgr6 is a protein integral to numerous physiological and pathological processes. While our understanding of its structure and function has advanced, there remain significant questions that warrant further exploration. These include the specific signaling pathway of Lgr6, its precise roles in various pathological and physiological processes, and how Lgr6 can be leveraged for disease diagnosis and treatment. Understanding these questions will deepen our insight into Lgr6's biological importance and lay a theoretical groundwork for its practical medical use.

The extracellular LRR domain of Lgr6 can bind to several ligands. RSPOs enhance Wnt/β-catenin signaling by binding to the LRR domain of Lgr6.^15^ Maresin-1 (MaR1), working as a lipid mediator, activates the non-canonical GPCR-cyclic adenosine monophosphate (cAMP) pathway through Lgr6.^11^^,^^16^ The mechanisms of the two are different. RSPOs synergistically amplify Wnt signaling with Lgr6/LRP6 complex (pro-tumorigenic)^17^^,^^18^; MaR1-Lgr6 inhibits Ca^2+^ influx/calcium/calmodulin-dependent kinase II (CaMKII) via cAMP and activates Nrf2/HO-1 pathway (vasoprotective).^19^^,^^20^ In the tumor microenvironment, RSPOs may suppress the anti-inflammatory effect of MaR1 (such as lung cancer Wnt overactivation).^18^^,^^21^ In the kidney, MaR1-Lgr6 predominates anti-oxidation independent of the Wnt pathway.^22^

## Expression of Lgr6 in different systems

### Urinary system

**Figure 3:**
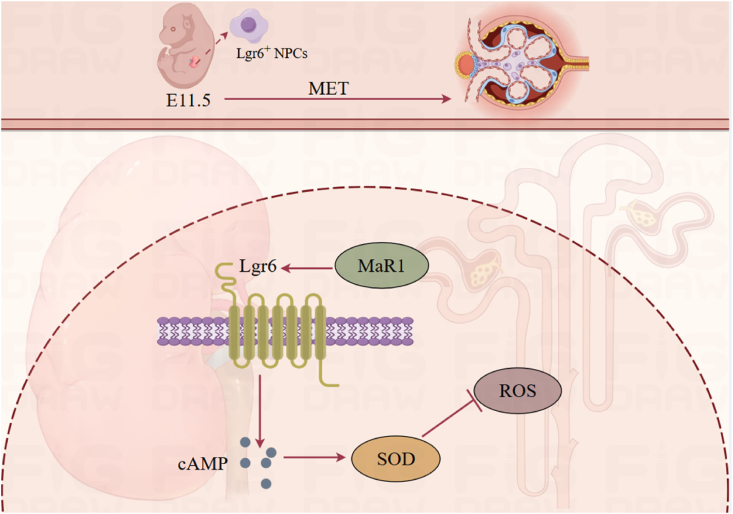
Role of Lgr6 in kidney development and inflammation. MET, Mesenchymal-to-epithelial transition; MaR1 (Maresin 1); SOD, Superoxide dismutase; ROS, Reactive oxygen species; Camp, Cyclic adenosine monophosphate; NPCs, Nephron progenitor cells.

Lgr6 marks nephron progenitor cells and is essential for kidney development (Fig. 3). The expression of Lgr6 commences at embryonic day 11.5, signifying the transition from nephron progenitor cells to mesenchymal-to-epithelial transition, and contributes to early nephrogenesis, exhibiting a unique expression profile compared with Lgr4/5.^14^ Notably, Lgr4, but not Lgr5/6, regulates RSPO signaling in nephron progenitors.^14^

Only some Lgr6 markers identify sine oculis homeobox homolog 2 (SIX2)-expressing Cap mesenchyme cells with nephrogenic potential.^2^ Imaging and single-cell RNA sequencing indicate that Lgr6^+^ cells can differentiate into tubular epithelium and podocytes.^2^ Consequently, Lgr6^+^ Cap mesenchyme cells may respond more readily to Wnt signaling, influencing their lineage commitment.^2^ Consequently, Lgr6-positive Cap mesenchyme cells may exhibit heightened susceptibility to Wnt-induced signals, which subsequently dictate their lineage formation.^23^ This indicates that Lgr6 is not directly involved in nephrogenesis, but rather its expression marks stem cells. Alternatively, there may be redundant regulatory pathways, such as co-expression of WNT4.^24^

In contrast to LGR4 and LGR5, Lgr6 marks early nephron progenitor cells capable of developing into diverse nephrogenic lineages.^25^ Moreover, Lgr6^+^ cells can generate all mesenchymal-derived glomerular segments from embryonic stages through postnatal life via mesenchymal-to-epithelial transition, thus representing a bona fide renal unit precursor cell population with regenerative potential.

Lgr6 expression in the kidneys is low in diabetic kidney disease, which can be reversed by MaR1. MaR1, a lipid mediator with both anti-inflammatory and pro-resolving capabilities, promotes tissue repair and alleviates pain.^9^ Maresin-induced macrophage activation stimulates phagocytosis and alleviates inflammatory pain by altering cytokine production towards an anti-inflammatory profile.^9^ Lgr6 is one of the main receptors for MaR1 and protects against various diseases. Prior research indicates that MaR1 ameliorates diabetic kidney disease via reactive oxygen species (ROS) reduction, suppressed inflammation, and diminished fibrosis.^10^

Lgr6, one of the receptors for MaR1, has been shown to up-regulate cAMP expression upon activation.^11^ The increased cAMP expression subsequently leads to an increase in superoxide dismutase 2 (SOD2).^12^ cAMP functions via Lgr6,^13^ enhancing SOD2 activity in an Lgr6-dependent manner.^13^^,^^26^ In diabetic kidney disease models, MaR1 activated the Lgr6/cAMP/SOD2 signaling pathway. This axis inhibited ROS-driven inflammation under hyperglycemia.^22^

### Respiratory system

**Figure 4:**
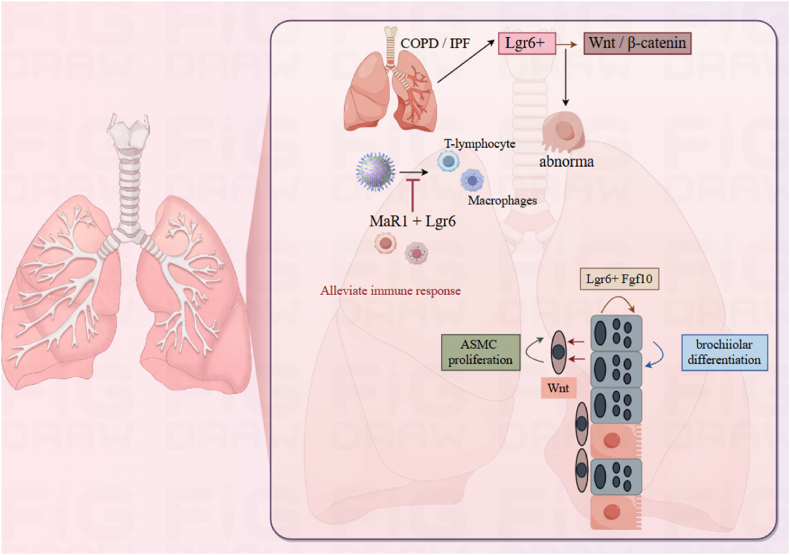
Role of Lgr6 in respiratory physiology and pathologies. COPD, Chronic obstructive pulmonary disease; IPF, Idiopathic pulmonary fibrosis; Wnt, Wingless/Integrated; ASMC, Airway smooth muscle cell; Fgf10, Fibroblast Growth Factor 10.

Lgr6 serves as a key stem cell marker in the lungs, playing a crucial role in maintaining the homeostasis of lung epithelial tissue and the process of injury repair (Fig. 4). Unlike Lgr5 cells, which are distributed throughout the alveoli, Lgr6^+^ cells predominantly inhabit the bronchial epithelium and within the alveolar space. These cells can guide Scgb1a1 lineage cells to differentiate directly into airway cells towards Lgr5^+^ cells. The Lgr6^+^ cell group forms a subset of smooth muscle cells surrounding the airway's lining, which helps in the direct transformation of epithelial stem cells due to the synergy between Wnt and fibroblast growth factor-10 (Fgf10) pathways. Knocking out the gene of Lgr6 cells hinders the repair of airway damage *in vivo*.^27^ The E-Cad/Lgr6 population, putative stem cells isolated from human lungs, contributes to lung homeostasis maintenance. These cells have self-renewal capacity and potential for ex vivo and *in vivo* differentiation.^28^ E-Cad/Lgr6 population is important for lung homeostasis and repair after injury. In human lung, Lgr6+ stem cells (LAPs) receive p38α input to make stromal cell-derived factor 1 (SDF-1), which in turn initiates the repair process and activates the niche.^29^ P38α plays a pivotal role in kickstarting fibroblast activation and cytokine release, especially in the case of tumor necrosis factor alpha (TNFα). This intercellular signaling network initiates a chain reaction, attracting endothelial cells and setting the stage for a functional microenvironment. When this communication line gets disrupted, it can throw a wrench in the proper differentiation of lung stem cells in the body, which might ultimately result in respiratory issues and illnesses. Additionally, epithelial progenitor cells expressing Lgr6 show a significant increase in the expression of senescence-associated markers in areas of abnormal regeneration, chronic injury, and fibrosis.^30^

In non-small cell lung cancer, Lgr6 is restricted to tumor areas, undetected in nearby normal tissue, and increases with disease advancement.^21^ As non-small cell lung cancer malignantly progresses, the level of Lgr6 expression escalates, particularly in various stages of lung adenocarcinoma. In this scenario, Lgr6^+^ cells showcase a more significant potential for tumor formation and an increased ability to maintain their original state in contrast to Lgr6^–^ cells.^21^ This discrepancy suggests an imbalance in the p38α and miR-17-92 pathways. When the p38α signaling pathway falls short, it causes widespread mismanagement of Wnt signaling pathway elements, which in turn encourages the suppression of Wnt inhibitors and strengthens their activators.^31^^,^^32^ The Wnt signaling pathway regulates stem cell maintenance and has a key role in many cancers.^33^ When p38α is absent, it amplifies the activity of low-density LRP6. This protein works hand in glove with RSPO receptors like Lgr6 to turbocharge Wnt signaling, effectively fueling uncontrolled cell proliferation.^17^^,^^34^ Additionally, Lgr6, as a promoter of Wnt/β-catenin signaling transduction, enhances the response to Wnt ligands and amplifies receptor signals,^18^^,^^35^ leading to the specific survival of Lgr6^+^ cells during cancer progression. Overexpression of aquaporin 5 (AQP5) in Lgr6^+^ tumor cells further underscores the high tumorigenicity of Lgr6^+^ cells.^31^^,^^36^ Upon activation of the Wnt/β-catenin pathway, Lgr6+ cells retain their tumorigenic potential but exhibit a loss of differentiation capacity. They shed epithelial cell (E-cadherin) characteristics and acquire stem cell (Lgr6) markers..^37^^,^^38^

Lgr6 is also highly expressed in dysplastic lung progenitors in COPD and IPF. In areas of dysregulated regeneration, ongoing destruction and fibrosis, Lgr6+ epithelial progenitors have increased expression of senescence-related markers.^30^ This indicates a role for Lgr6 in chronic activation of Wnt/β-catenin signaling, leading to injury and depletion of epithelial stem cells in COPD and IPF.^39^ Lgr6 localizes to basal, spherical and alveolar type II precursor cells in small airway bronchioles with fibrosis and to narrowed airways with loss or extension of bronchial cells into the alveolar compartment. Lgr6+ cells are significantly increased in lung biopsies from patients with COPD and IPF versus donor tissue.^39^

Lgr6 signaling is important in regulating the number and function of regulatory T cells (Tregs) during respiratory syncytial virus (RSV) infection.^40^ The signaling mediated by Lgr6 is instrumental in modulating the quantity and functionality of Tregs. MaR1 restores RSV type 2 Treg suppression by alleviating viral lung inflammation and mucosal cell metaplasia; the MaR1 receptor, Lgr6, is involved,^11^ and is constitutively highly expressed on Tregs and macrophages, with a significant alteration in Lgr6 expression in Tregs observed during RSV infection. Macrophages are a key producer of MaR1,^41^ and Lgr6's continuous presence on macrophages aids in self-regulating their response to reduce.^11^

Research indicates that Lgr6-knockout mice really get hit hard by RSV infections, showing a more jacked-up type 2 immune response. We are talking about a serious uptick in mucous cell metaplasia, a spike in gob5 transcription, and interleukin-13 (IL-13) protein levels going through the roof. On top of that, you see a surge in IL-13-producing CD4 T cells and innate lymphoid cells.^40^ Lgr6 significantly contributes to RSV infection's immune response through its interaction with the MaR1 pathway, vital for modulating Tregs' function and count. The absence of Lgr6 exacerbates inflammation and increases viral load, while MaR1 exerts immunomodulatory effects by activating the Lgr6 receptor.

### Skeletal system

**Figure 5:**
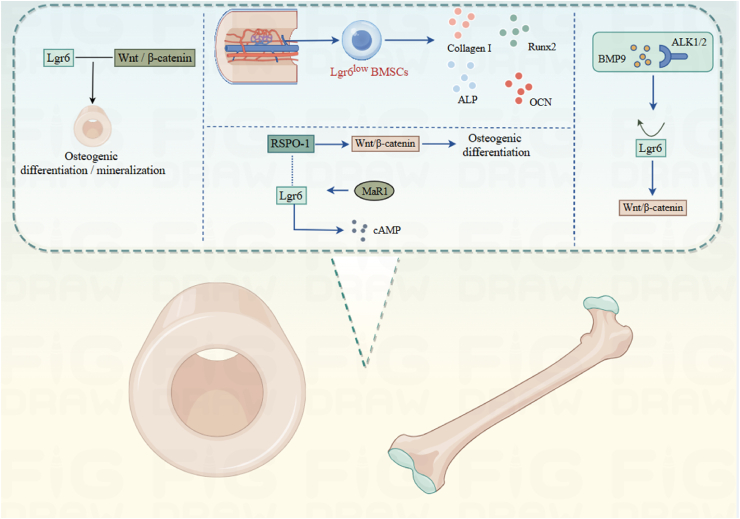
Role of Lgr6 in bone formation and injury. BMSCs, Bone marrow stromal cells; BMP9, Bone morphogenetic protein 9; ALK1/2, Activin receptor-like kinase 1/2; ALP, Alkaline phosphatase; OCN, Osteocalcin; RSPO-1, R-spondin 1; Runx2, Runt-related transcription factor.

In bone, Lgr6 has been pegged as a marker for osteoprogenitor cells in mice and shows a fluctuating expression pattern as mesenchymal stem cells (MSCs) differentiate into bone cells *in vitro*, hinting that it plays a role in bone formation.^42^^,^^43^ These observations are consistent with human osteogenesis, where Lgr6 also demonstrates dynamic expression during the *in vitro* osteogenic differentiation of human MSCs and osteoblasts.^44^^,^^45^ Notably, the present findings confirm the important role of Lgr6 in bone homeostasis and repair (Fig. 5). Specifically, Lgr6 plays an essential role in digit regeneration in mice. A targeted gene sequencing study of postmenopausal Chinese women has revealed that Lgr6 is associated with osteoporosis.^42^^,^^46^ Additionally, Lgr6 exhibits varying expression throughout the differentiation of skeletal stem progenitor cells (SSPCs) originating from diverse mesenchymal sources, such as skull, periosteum, and bone marrow.^43^^,^^47^^,^^48^ In humans, polymorphisms of the Lgr6 locus are associated with osteoporosis.^46^

Lgr6 is transiently up-regulated in osteoprogenitor cells post-fracture damage.^47^ Bone marrow-derived MSCs from hip fracture patients show Lgr6 as one of the most highly up-regulated genes after injury.^49^ Moreover, Lgr6 is essential for preserving bone mass and enabling efficient postnatal bone repair in adult mice.^47^^,^^48^ More specifically, mice with the Lgr6 knockout (or Lgr6-null) exhibit less bone mass and a weaker capacity to heal fractures. These animals' osteogenic precursor cells do not form colonies as effectively, and their ability to differentiate into bone-forming cells is notably diminished. This is linked back to a weakened Wnt/β-catenin signaling pathway.^47^ When these mice sustain a fracture, their periosteal progenitor cells fail to multiply as they should, and their alkaline phosphatase levels drop. Additionally, their natural bone formation and mineralization are severely impeded.^47^ On the other hand, Lgr6 boosts bone formation and mineralization by triggering the Wnt/β-catenin signaling cascade. More precisely, it reinforces Wnt signaling through β-catenin stabilization, which in turn drives the osteogenic differentiation and mineralization process in MC3T3-E1 cells.^48^^,^^50^ On the flip side, suppressing Lgr6 expression disrupts osteogenic differentiation and mineralization by accelerating β-catenin breakdown. This impairment effectively shuts down the Wnt/β-catenin signaling cascade.

In bone marrow stromal cells (BMSCs), the suppression of Lgr6 expression enhances osteogenic differentiation, thereby facilitating fracture healing. Empirical evidence indicates that BMSCs with diminished Lgr6 expression exhibit superior osteogenic potential compared with those with normal Lgr6 levels. Moreover, suppressing Lgr6 triggers an upsurge in the synthesis of osteogenesis-related proteins, including angiotensin II (AngII), collagen I, Runx2, and osteocalcin (OCN). Conversely, Lgr6's overexpression leads to a drop in these protein concentrations. Studies hint that BMSCs with diminished Lgr6 activity exhibit enhanced expression of collagen I, Runx2 (the runt-related transcription factor 2), and OCN, along with alkaline phosphatase, which is also known as alkaline phosphatase. These cells also display a notably greater level of *in vitro* mineralization. This evidence suggests that Lgr6's underexpression is pivotal in driving the osteogenic differentiation of BMSCs in a laboratory setting.^45^ Moreover, transplanting Lgr6-knockout BMSCs improves fracture healing *in vivo*, presenting new clinical approaches for bone regeneration.^45^

Studies indicate that Lgr6 is closely linked to the bone morphogenetic protein (BMP) signaling pathway.^51^^,^^52^ The function of Lgr6 in osteogenesis markedly influences the Bmp signaling pathway by modulating the cWnt signaling pathway.^53^, ^54^, ^55^ Lgr6 is instrumental in achieving peak bone mass and orchestrates bone formation through differential ligand utilization.^48^ Receptor activator of nuclear factor kappa B ligand (RANKL) can prompt the differentiation of osteoblasts into osteoclasts, which are implicated in bone resorption and destruction. Consequently, Lgr6 may safeguard bone tissue from excessive damage by curtailing the activity of RANKL.

Furthermore, the expression of Lgr6 and RSPO1 is found to overlap in late-stage osteoarthritis samples. RSPO1 is recognized for its role in promoting the differentiation process of osteoblasts via the WNT signaling pathway.^52^ The detection of RSPO1 and Lgr6 expression during osteoblast differentiation suggests that Lgr6 may function as a receptor for RSPO1, thereby mediating its impact on WNT signal stimulation. Other research indicates that MaR1 is linked to a synthetic metabolic bone phenotype.^56^ Lgr6 has been demonstrated to interact with MaR1, thereby potentiating G protein-coupled signaling and cAMP levels.^48^ This indicates that the Lgr6 receptor might employ alternative signaling mechanisms, beyond Wnt and Bmp, to control bone formation.

Bioinformatics studies identified Lgr6 as a key gene closely linked to the progression of intervertebral disc degeneration, where it appears to function as a protective factor.^57^ At the molecular level, Lgr6 preserves disc homeostasis through a dual mechanism: it modulates both immune function and tissue regeneration. On one hand, it boosts macrophage activity by increasing phagocytic receptor expression (MERTK, AXL, TYRO3, CX3CR1), enhancing their ability to clear apoptotic nucleus pulposus cells. On the other hand, it maintains extracellular matrix balance by stimulating collagen type II alpha 1 chain (COL2A1) production while suppressing matrix metallopeptidase 13 (MMP13) expression. Additionally, Lgr6 exerts anti-apoptotic effects by regulating cell death proteins, namely, up-regulating B-cell lymphoma 2 (BCL2) while down-regulating cleaved caspase 3 and BAX.^57^^,^^58^

### BMP and Wnt signaling in Lgr6-mediated bone regeneration

Lgr6, a marker for mouse bone progenitor cells, exhibits a fluctuating expression pattern as MSCs differentiate into bone cells *in vitro*, suggesting that it plays a pivotal role in bone development.^42^^,^^43^ This expression behavior appears to hold true in human osteogenesis as well. In human MSCs and osteoblasts, Lgr6 demonstrates comparable dynamic shifts during *in vitro* osteogenic differentiation.^44^^,^^45^ Furthermore, current research indicates that Lgr6^+^ cells are instrumental in bone regeneration, orchestrating this process through the combined regulation of the Wnt/β-catenin and BMP signaling pathways.

Lgr6 boosts the intensity of Wnt/β-catenin signaling by binding to RSPOs.^15^ During bone repair, a temporary increase in Lgr6 levels revs up the Wnt pathway, causing β-catenin to move into the nucleus. This triggers the growth and development of osteogenic stem cells.^47^^,^^50^ Mice lacking Lgr6 show less bone density and a longer healing time for fractures, a result of the weakened Wnt/β-catenin signaling. This signaling's disruption, which speeds up the breakdown of β-catenin, hampers the differentiation and mineralization of osteogenic cells.^47^^,^^48^

Lgr6 exhibits a strong functional interplay with the BMP signaling cascade. BMP2 triggers the expression of osteogenic markers like Runx2 and OCN via Smad1/5/8 phosphorylation, while Lgr6 potentiates BMP receptor activity, creating a self-reinforcing regulatory cycle.^51^, ^52^, ^53^ During initial fracture repair, BMP2/6 works in concert with the Lgr6-RSPO complex: BMP signaling primarily governs bone matrix formation and mineralization, whereas Wnt signaling preserves the progenitor cell population. This spatiotemporal synergy between the two pathways facilitates callus maturation.^52^^,^^54^ Notably, in advanced osteoarthritis specimens, researchers detected co-localization of Lgr6 and RSPO1, implying that Lgr6 may serve as an RSPO1 receptor to modulate bone remodeling through WNT-BMP pathway crosstalk.^52^ In vitro suppression of Lgr6 in BMSCs significantly enhances their osteogenesis capacity, and *in vivo* transplantation of Lgr6 knockout BMSCs accelerates fracture healing.^45^

### Genital system

**Figure 6:**
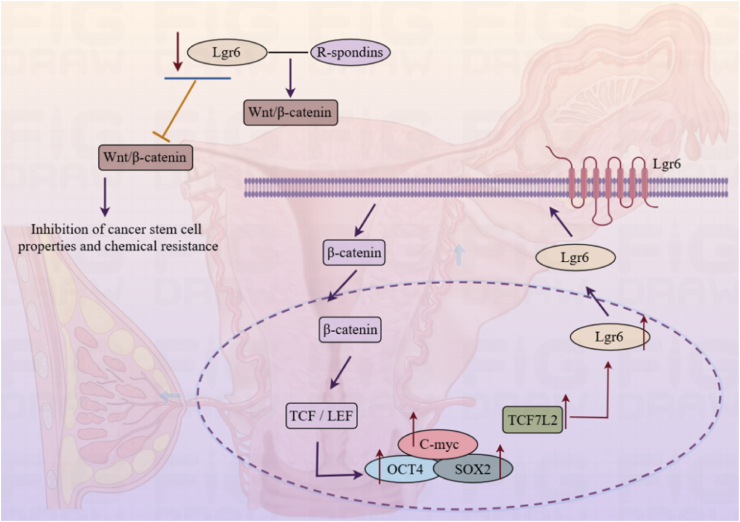
The involvement of Lgr6 in the genital system. Wnt, Wingless/Integrated; TCF, T-cell factor; LEF, Lymphoid enhancer-binding factor; C-myc, Myelocytomatosis viral oncogene homolog; OCT4, Octamer-binding transcription factor; SOX2, SRY-Box Transcription Factor 2; TCF7L2, Transcription factor 7-like 2.

Lgr6, as a key stem cell marker in the reproductive system, regulates the development and maintenance of gonadal stability, and its abnormal expression is associated with pathological processes such as tumors in the reproductive system (Fig. 6). Studies have shown that the level of Lgr6 is higher in ovarian cancer cells. Additionally, an excess of Lgr6 correlates with a less favorable reaction to chemotherapy in patients battling this illness.^59^^,^^60^ The enhancement of Wnt signaling by Lgr6 contributes to the development and progression of high-grade serous ovarian cancer.^61^ Lgr6 protein has been demonstrated to enhance the Wnt/β-catenin signaling pathway's intensity by interacting with RSPOs.^15^ Substantial reduction of Lgr6 expression can significantly impede the Wnt/b-catenin signaling cascade in ovarian cancer cells, effectively suppressing the attributes of cancer stem cells and chemoresistance.^62^

Lgr6 is a key player in revving up the Wnt/β-catenin signaling route, causing a bump in protein levels of pivotal stem cell factors related to pluripotency, like SRY-box transcription factor 2 (SOX2) and octamer-binding transcription factor 4 (OCT4). This boost is especially noteworthy in cervical cancer stem cells that are chock-full of Lgr6 activity.^63^ Cells overexpressing Lgr6 showed increased selfrenewal ability and tumorigenic capacity.^63^ Signal transducer and activator of transcription 2 (TCF7L2) is a key transcription factor in the WNT signaling cascade.^64^ TCF7L2 serves as a mediator in the subsequent stages of Wnt signaling, effectively interacting with β-catenin.^65^ Once it binds to nuclear β-catenin, this factor revs up the expression of Lgr6, effectively spurring the Wnt signaling cascade further and setting up a positive feedback loop. In cervical cancer, TCF7L2 gives a leg up to the expression of Lgr6 by latching onto its promoter directly.^63^

Lgr6 is also implicated in marking breast cells,^23^ with Lgr6 labeling a rare population of basal and luminal components within mouse mammary glands.^5^ T cells flagged with Lgr6 can derive from luminal breast cancer origins.^62^ Genome-wide analyses link Lgr6 to estrogen (ER)-negative breast cancer risk.^66^^,^^67^ In adulthood, Lgr6^+^ cells regain their proliferative capacity when pregnant or stimulated by ovarian hormones. Their descendants form acini during multiple pregnancies. Carcinogenic mutations in Lgr6^+^ cells drive luminal cell expansion, culminating in breast tumor development.^5^ Lgr6 identifies mammary progenitors that initiate tumors and sustain essential luminal breast cancer cells.

### Nervous system

**Figure 7:**
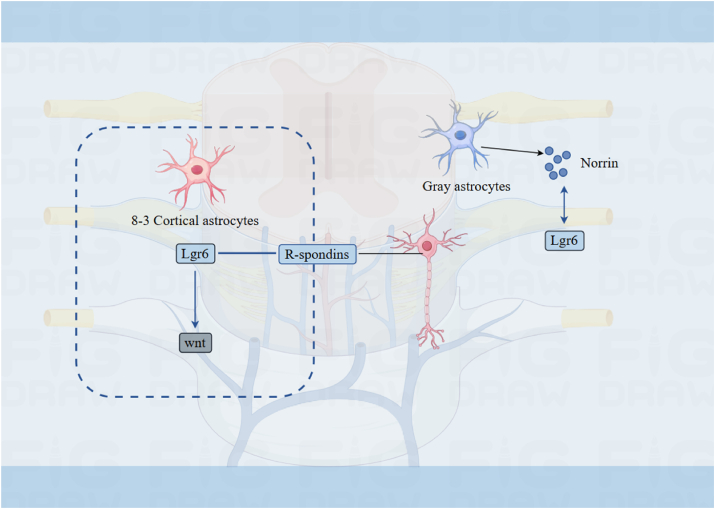
The involvement of Lgr6 in the nervous system. Wnt, Wingless/Integrated.

Lgr6 plays a key physiological function in regulating neuronal synapse plasticity and has therapeutic potential (Fig. 7). Research suggests that the expression of Lgr6 in keratin-forming cells is contingent upon skin innervation.^68^ Lgr6 has been widely demonstrated to have astrocyte specificity in the central nervous system.^69^^,^^70^ Lgr6 mediates Wnt signaling, a pathway highly active in gray matter astrocytes. Its ligand, RSPO1, is selectively secreted by pyramidal neurons.^23^^,^^68^^,^^71^ Lgr6 expression is associated with the distinct profile of the 8.3-astrocyte subset, predominantly found in the cortex's fifth layer. This region is also highly enriched with 8.3 astrocytes. Furthermore, the expression level of Lgr6 in 8.3-astrocytes is significantly higher than in other types of glial cells and neurons.^72^

Lgr6 has a notable connection with the protein Norrin, which is primarily produced in cortical astrocytes. Interestingly, this protein shows a strong overlap in its presence with Lgr6, particularly within the fifth layer of the cerebral cortex. This co-localization pattern is consistently observed in both mice and humans, highlighting a shared biological mechanism across species.^69^^,^^70^ Given that Norrin is exclusively released by 8.3-astrocytes, it holds potential as a therapeutic agent for modulating the dendritic spines and synapses of neurons.

### Digestive system

**Figure 8:**
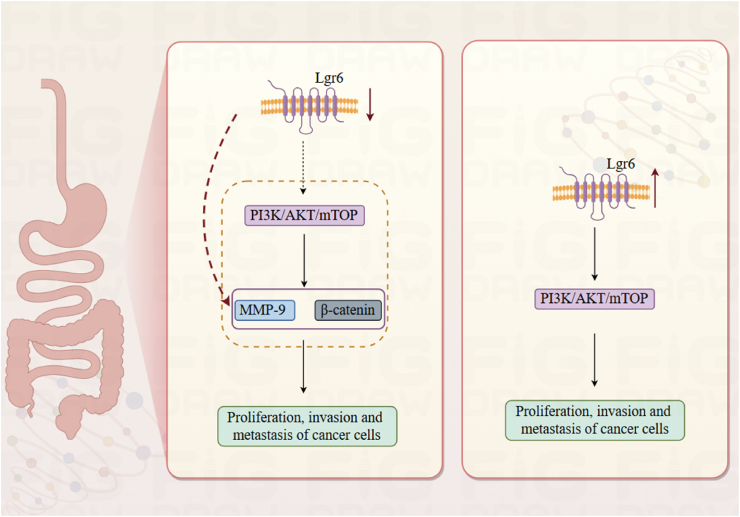
Role of Lgr6 in gastric and rectal cancer. PI3K, Phosphatidylinositol 3-kinase; AKT, Protein kinase B; mTOP, Mammalian target of rapamycin; MMP-9, Matrix metallopeptidase 9.

Mutations in Lgr6 have been identified in colorectal cancer, positioning Lgr6 as a potential candidate gene for this disease.^8^ Existing research indicates that Lgr6 is involved in several crucial cellular processes within colorectal cancer tissues, such as proliferation, invasion, and metastasis (Fig. 8).^15^ The PI3K/AKT pathway, implicated in cancers such as colorectal cancer, directly impacts cellular growth, migration, and invasion.^73^, ^74^, ^75^ Research has also shown that Lgr6 impacts the growth and infiltration of colorectal cancer cells through its regulation of the PI3K/AKT signaling cascade.^73^^,^^74^^,^^76^, ^77^, ^78^ Up-regulation of Lgr6 expression has been confirmed in diseases ranging from gastric carcinoma^79^ to basaloid skin tumors,^80^ indicating its promise as both a predictive biomarker and a therapeutic target in advanced colorectal cancer. Furthermore, high levels of Lgr6 mRNA in lymph nodes have been associated with a shorter disease-free survival period, indicating its potential as a supplementary biomarker to carcinoembryonic antigen (CEA) and CXC chemokine ligand 16 (CXCL16) for detecting postoperative recurrence in colon cancer patients.^81^

It has been reported that Lgr6 expression is very low in normal human esophageal tissue, but high in the esophageal cancer tissues of patients.^82^ Similarly, Lgr6 expression is significantly elevated in gastric cancer cell lines and tissues.^79^ Moreover, Lgr6 is identified as a facilitator of gastric cancer advancement via the PI3K/AKT/mammalian target of rapamycin (mTOR) signaling pathway.^83^ Research has shown that when Lgr6 expression is low, it throws a wrench into the expression of proteins that have a hand in cell migration, like MMP9 and β-catenin. This, in turn, really puts the brakes on the proliferation and invasive behavior of gastric cancer cells.^84^ It is known that Twist1, a Wnt target and epithelial-mesenchymal transition marker, is related to Lgr6.^85^^,^^86^ Lgr6 triggers epithelial-mesenchymal transition through Twist1.^87^

Studies indicate that WNT activation elevates Lgr6 expression. Conversely, inhibition of WNT is associated with decreased levels of Lgr6 expression. Therefore, the regulation of Lgr6 in pancreatic ductal adenocarcinoma may constitute a key positive feedback loop in the WNT signaling activity circuit.^3^

### Cardiovascular system

Lgr6 serves as a pivotal receptor for the pro-resolving mediator MaR1, significantly influencing the hypertensive vascular remodeling process.^11^ It has been shown that the serum level of MaR1 is significantly reduced in patients with hypertension and negatively correlated with systolic blood pressure.^19^ In an AngII-induced hypertension model, the deletion of the Lgr6 gene amplifies blood pressure spikes, exacerbates structural damage, such as vascular wall thickening, fibrosis, and aortic dilation, and intensifies the phenotypic transformation of vascular smooth muscle cells (with a drop in contraction markers like α-SMA/SM22α and a rise in synthetic markers like osteopontin), as well as promotes cell pyroptosis (triggered by NLRP3/IL-1β/IL-18).^19^ The core mechanism involves Lgr6 facilitating the inhibition of Ca^2+^ influx and reducing CaMKII phosphorylation,^20^ thereby kickstarting the Nrf2/HO-1 pathway and ultimately quelling vascular smooth muscle cell proliferation, migration, and pyroptosis. It is worth noting that without Lgr6, the protective effects of MaR1 on the vasculature vanish, but the CaMKII inhibitor KN-93 can partially mitigate this detrimental effect, solidifying Lgr6's crucial role as the receptor for MaR1 in maintaining vascular homeostasis. This novel pathway opens up new possibilities for GPCR targeting in hypertension treatment.^88^

In pulmonary arterial hypertension, paradoxically, early clinical and animal studies demonstrated that patients with pulmonary arterial hypertension had decreased serum MaR1 levels, and the pulmonary arterial hypertension mouse model also showed decreased serum MaR1 with down-regulation of Lgr6 protein expression in lung tissues.^89^ The underlying molecular mechanisms revealed that Lgr6 enabled MaR1 to inhibit STAT3, AKT, extracellular signal-regulated kinase (ERK), and forkhead box transcription factor O1 (FOXO1) phosphorylation, thereby suppressing the proliferation and migration of pulmonary artery smooth muscle cells and promoting apoptosis.^11^^,^^89^

However, recent studies have found that in the pulmonary arterial hypertension models induced by monocrotaline and hypoxia, there is a significant up-regulation of Lgr6 protein expression in lung tissues, which is positively correlated with the severity of the disease.^42^ This discovery is significantly different from the protective role of LGR6 advocated by early studies,^11^^,^^88^ indicating that the functional role of Lgr6 in pulmonary arterial hypertension still needs to be further verified through gene knockout models.^42^

Lgr6 is crucial in safeguarding the heart against damage from ischemia-reperfusion situations. Research indicates that post-injury, Lgr6 levels plummet in cardiac tissue, spanning across non-ischemic, border, and ischemic areas, and across several cardiomyocyte lines like mouse HL1, rat H9C2, and human AC16.^89^ Knocking out Lgr6 in mice led to a worsened heart attack, marked by larger areas of heart muscle death, higher levels of cardiac enzymes like cardiac troponin T (cTnT), creatine kinase MB (CK-MB), and lactate dehydrogenase (LDH), and a propensity for necroptosis over apoptosis, as evidenced by the rise in phosphorylated receptor-interacting serine/threonine-protein kinase 1 (RIPK1), RIPK3, and mixed-lineage kinase domain-like pseudokinase (MLKL).^90^ Conversely, boosting Lgr6 in cardiomyocytes significantly lessened the damage.^91^ Mechanistically, Lgr6 kickstarts the Wnt signaling pathway by binding to RSPO3, thus quelling the transcription factor STAT2. STAT2 then binds to and turns on the Z-DNA binding protein 1 (ZBP1) promoter, creating a STAT2-ZBP1 axis that powers necroptosis.^92^^,^^93^ Halting STAT2 or ZBP1 can undo myocardial necrosis in the absence of LGR6.^94^^,^^95^ Furthermore, RSPO3-triggered Lgr6 activation mirrors the protective effects, diminishing ischemia-reperfusion injury.^95^ Furthermore, RSPO3-triggered Lgr6 activation mirrors the protective effects, diminishing ischemia-reperfusion injury.^96^^,^^97^ In summary, the RSPO3-LGR6-STAT2-ZBP1 signaling axis regulates necroptosis and becomes a potential therapeutic target for myocardial ischemia-reperfusion injury.^98^

Lgr6 alleviates ferroptosis in diabetic cardiomyopathy by regulating mitochondrial biogenesis. It has been found that Lgr6 expression is significantly up-regulated in diabetic hearts and in high glucose-treated HL1 cardiomyocytes.^99^ Functionally, L6KO exacerbates cardiac dysfunction and remodeling in diabetic mice. In terms of function, knocking out L6KO makes the heart's issues and reshaping in diabetic mice worse^100^; however, giving cardiomyocytes a boost of LGR6 (via AAV9-cTnT-LGR6) has been shown to significantly improve those problems.^101^ When Lgr6 is more abundant, it stops STAT3 from getting phosphorylated and from moving into the nucleus, thus taking the brakes off STAT3's ability to repress the crucial mitochondrial biogenesis factor, peroxisome proliferator-activated receptor gamma coactivator-1 alpha (Pgc1a), ultimately fostering the growth of mitochondria.^102^^,^^103^ The STAT3 inhibitor S31-201 or the Pgc1a agonist ZLN005 reverses the mitochondrial dysfunction and ferroptosis induced by L6KO.^104^^,^^105^ Furthermore, turning on the RSPO3-LGR6 pathway can mimic the heart-protecting effects and help with dilated cardiomyopathy,^39^^,^^106^ and this protective effect is all thanks to the cAMP/EPAC1 signaling that keeps STAT3 in check.^107^^,^^108^ In summary, the LGR6-STAT3-Pgc1a signaling axis provides a new therapeutic target for diabetic cardiomyopathy by coordinating mitochondrial biogenesis with ferroptosis inhibition. In a nutshell, the LGR6-STAT3-Pgc1a pathway is a fresh angle for treating diabetic cardiomyopathy, as it balances out mitochondrial biogenesis with the suppression of ferroptosis.^109^^,^^110^

Knocking down Lgr6 specifically in heart muscle cells worsened cardiac hypertrophy, impaired heart function, and messed with metabolism. On the flip side, boosting Lgr6 expression eased these problems significantly.^90^^,^^111^ Lgr6 seemed to crank up the production of ubiquitin-specific protease 4 (USP4), a deubiquitinating enzyme, by kicking the cyclic guanosine monophosphate (cGMP)/protein kinase G (PKG)/cAMP responsive element binding protein 1 (CREB1) signaling pathway into gear. This, in turn, helped stabilize peroxisome proliferator-activated receptor α (PPARα) by removing ubiquitin tags from it. Ultimately, this whole cascade counteracted the metabolic mayhem caused by pressure overload in heart muscle cells. It put the brakes on glycolysis while boosting fatty acid oxidation and oxidative phosphorylation, getting myocardial energy levels back on track.^112^^,^^113^ Interestingly, MaR1, a drug that selectively activates Lgr6, softened cardiac hypertrophy by firing up the Lgr6/USP4/PPARα pathway, suggesting a possible new angle for treating heart problems in the clinic.^90^ Taking everything together, the Lgr6-USP4-PPARα signaling pathway is shaping up as a promising target for treating unhealthy heart enlargement by tweaking how the heart muscle cells handle energy.^114^^,^^115^

### Skin

**Figure 9:**
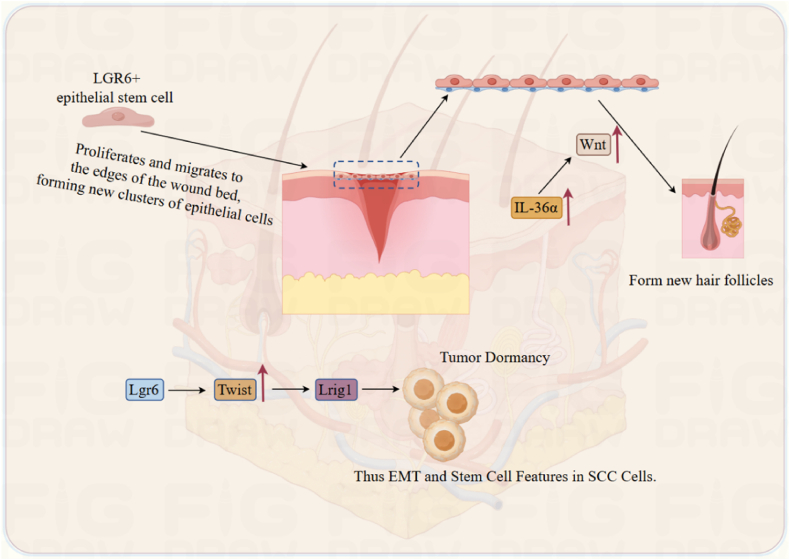
The involvement of Lgr6 in skin. EMT, Epithelial-mesenchymal transition; SCC, Squamous cell carcinoma; Wnt, Wingless/Integrated.

LGR6, as a marker of skin stem cells, plays a key role in skin injury repair, hair follicle regeneration, and skin homeostasis (Fig. 9). Lgr5 and Lgr6-expressing stem cells rank among the most primitive in epidermal development, fundamentally contributing to the maintenance of adult skin homeostasis.^4^^,^^23^^,^^116^ Lgr6 was identified as a molecular marker for stem cells located in the isthmus of hair follicles, which were proposed to give rise to the hair follicle (HF), sebaceous gland (SG) and interfollicular epidermis (IFE) lineages.^23^ Emerging research indicates the presence of Lgr6 within the basal cells of both the IFE and the SG.^68^^,^^117^ New research indicates that Lgr6^+^ cells constitute a durable, self-sustaining population within every adult skin region. They can be pinpointed in the hair follicle's isthmus, the sebaceous glands, and the interfollicular epidermis, owing to sophisticated multicolor lineage tracing methods.^4^

Lgr6^+^ epithelial stem cells, situated close to a wound bed, multiply and travel to the wound perimeter, creating fresh aggregations of epithelial cells.^23^ The inflammatory cytokines in wounded skin boost stem cell migration to the injury, accelerating epithelial regeneration.^118^, ^119^, ^120^ These Lgr6^+^ epithelial stem cells, often regarded as the most rudimentary type of epidermal stem cells, do not just excel at facilitating long-term wound healing; they also hold the remarkable ability to trigger the development of fresh hair follicles.^23^^,^^121^ Moreover, the studies indicate that such epithelial stem cells are governed by a Wnt-activated signaling route within the surrounding microenvironment.^121^ The application of Lgr6^+^ epithelial stem cells onto different acellular matrices significantly boosts the healing process by driving the migration, multiplication, and specialization of these cells, ultimately leading to the formation of functional skin that can generate new hair follicles.^7^ Moreover, when these stem cells are introduced into deep wounds where hair follicle bulges are absent, they not only regenerate hair follicles but also form cystic structures.^7^

A small population of Lgr6-positive cells has been found among immortalized keratinocytes, and these cells increase in frequency in late-stage squamous cell carcinoma (SCC). Lgr6 is also highly expressed in cells with stem cell characteristics. Reducing Lgr6 expression *in vivo* leads to increased epidermal cell proliferation and expanded lineage tracing of Lgr6-positive epidermal stem cells. Interestingly, Lgr6 knockout mice are more susceptible to SCC, which is associated with the compensatory upregulation of Lgr5.

Lgr6, a protein pivotal to skin regeneration and wound healing, has been found to enhance its expression through interaction with nerves in Lgr6-marked epidermal stem cells.^116^ This interaction up-regulates gene expression within the epidermal stem cell pool of these cells, primarily involving processes such as epithelial development, cell differentiation, regulation of cell adhesion, and morphogenesis. The loss of Lgr6 stem cells in the epidermis can slow down the healing process, and the desensitization of the skin might change how these stem cells contribute to healing. This interplay is crucial for keeping the Lgr6 epidermal stem cells in a prime position for swift growth.^116^ Inherited genetic mutations in Lgr6^+^ cells frequently lead to epithelial hyperplasia, sebaceous gland enlargement, and benign papillomas.^122^ IL-36α is capable of enhancing mediators in the Wnt/β-catenin pathway, which is a bridge between the β-catenin and IL-6/STAT3 signaling networks. Lgr6, a molecule tied to IL-36α, has been found to foster the process of healing and even stimulate the formation of new hair follicles following an injury.^123^

The Lgr6 gene, closely associated with the Lgr5 stem cell gene, initiates expression during the initial phase of hair growth in mouse embryos. In adult hair, Lgr6^+^ cells are situated above the protruding portion of the hair follicles. During embryonic growth, Lgr6-positive cells play a pivotal role in shaping hair follicles, sebaceous glands, and the interfollicular epidermis. After birth, although these cells persist in producing sebaceous glands and interfollicular skin tissue, their role in hair follicle regeneration gradually declines over time. In adulthood, Lgr6^+^ cells contribute significantly to prolonged wound healing, even facilitating the regeneration of hair follicles. Research has confirmed that Lgr6 serves as a marker for the most fundamental epidermal stem cells. Implanting a scaffold infused with Lgr6^+^ epithelial stem cells into deep skin injuries has proven to accelerate the healing process, stimulate the formation of fresh hair follicles, and boost blood vessel growth.^7^

Lgr6 expression in the skin markedly correlates with neural innervation zones. Within the hair follicle, Lgr6 sticks close to the nerve endings and their paired Schwann cells throughout the entire lifecycle of the hair. Additionally, if you denude the skin, it triggers the Schwann cells to deteriorate, which in turn leads to a decrease in Lgr6 levels.^4^

Lgr6^+^ stem cells are crucial for epidermal development. During skin expansion, Lgr5^+^ cells and their progeny are predominantly localized within hair follicles, whereas Lgr6^+^ cells are distributed across both hair follicles and the dermal papillae. Following expansion, Lgr6-tdT cells are dispersed throughout all layers of the epidermis, encompassing the basal, spinous, and stratum corneum layers. Tension has been demonstrated to favor differentiation over self-renewal in Lgr6^+^ cells.^124^ In mature skin, Lgr5^+^ cells mainly populate the HF bulge, facilitating HF regeneration, whereas Lgr6^+^ cells are found in the IFE and HF zones of both the dorsal and ventral skin layers.^23^^,^^125^ Despite their closely overlapping developmental trajectories, these two cell types exhibit markedly different responses to tension. Interestingly, Lgr6 knockout mice exhibit an increased susceptibility to developing SCC. These data propose a model for individuals with inherited Wnt pathway gene variants, like RSPO1 or LGR4, showing increased susceptibility to squamous cell neoplasms.^126^

Lgr6 acts as a stem cell identifier in murine epidermal SCC,^87^^,^^127^ chiefly at the epithelial-mesenchymal transition zone within head-neck SCC, and its presence aligns with worsened prognosis.^128^ In mouse skin SCC, Lgr6 deficiency increases susceptibility to SCC formation, likely through compensatory Lgr5 up-regulation in Lgr6 absence,^127^ which results in increased Wnt activity that disrupts skin homeostasis. Twist1, a pivotal epithelial-mesenchymal transition marker, is instrumental in both the progression of ultraviolet B radiation-exposed SCC and the up-regulation of stem cell-related genes, leucine-rich repeats and immunoglobulin-like domains 1 (Lrig1) and Lgr6.^86^ Interestingly, Twist1, a gene regulated by Wnt signaling,^85^ is transcriptionally activated by Lgr6, which in turn triggers the epithelial-mesenchymal transition. Simultaneously, Twist1 up-regulates Lrig1, pushing tumor cells into a dormant state. This dual mechanism endows SCC cells with both epithelial-mesenchymal transition properties and stem-like features.^129^

Lgr6, a stem cell marker, contributes to the onset and advancement of multiple cancers. Studies have demonstrated that patients who are both human papillomavirus (HPV)-positive and Lgr6-positive exhibit a significantly higher survival rate compared with those who are Lgr6-negative, with their survival rate closely mirroring that of HPV-negative patients. This suggests a significant interaction between HPV, Lgr6, and RSPO2. The results suggest that Lgr6 could influence the malignancy of HPV-associated oral SCC and might act as a biomarker for distinguishing low-risk patient subsets.^129^

Activation of β-catenin in Lgr6^+^ epidermal stem cells may lead to varied effects, such as ectopic hair follicle formation and tumorigenesis. Given the association between different stem cells and various tumor types, as well as stromal reactions, compartmentalization of epidermal stem cells is fundamental to tumor heterogeneity.^117^ The study reveals that offspring stemming from Lgr6-positive stem cells can be found in the IFE, SG, and the higher part of the HF, which points to the fact that the collection of Lgr6-positive stem cells is consistently topping up a substantial segment of the adult epidermis.^117^ In the caudal IFE, ectopic hair follicles originating from Lgr6-expressing cells are primarily located in the interfollicular IFE, while the squamous IFE does not express Lgr6. This suggests that Lgr6 serves as a marker to differentiate between interfollicular and squamous stem cells.^130^

Research reveals Lgr6 as a marker for epidermal stem cells in mouse skin SCC.^127^ Despite Lgr6 being an active stimulator of Wnt signaling, its inactivation in somatic cells shows an inhibitory effect on the development of SCC. It is possible that the absence of the Lgr6 gene is causing the body to jack up the activity of other related pathways, sort of like a backup system stepping in to make up for the Lgr6's shortcomings. What's intriguing is that these mice without the Lgr6 gene are more prone to SCC. This research gives us a valuable framework to understand human patients with inherited mutations in Wnt pathway genes, like RSPO1 or LGR4, who are at a greater risk for developing squamous cell cancers.^131^

### Lgr6^+^ progenitors in tissue homeostasis and repair

Lgr6 orchestrates tissue-specific stem cell programs across organ systems, serving as a dynamic regulator of development, homeostasis, and regenerative responses. In the epidermis, Lgr6^+^ primitive stem cells reside within the hair follicle bulge/isthmus, SG, and IFE niches.^4^^,^^23^^,^^116^ These cells demonstrate multipotent differentiation capacity, migrating to wound edges to drive re-epithelialization, regenerate hair follicles, and accelerate healing through neovascularization in graft models.^7^^,^^23^^,^^121^ Crucially, their function is neuro-regulated: cutaneous nerve endings and Schwann cells sustain Lgr6 expression, while denervation impairs regenerative competence.^4^^,^^116^

Within pulmonary tissues, Lgr6^+^ bronchiolar/alveolar progenitors are spatially distinct from Lgr5^+^ alveolar cells.^27^^,^^28^ Following Lgr6 knockout, the airway exhibits impaired repair.^27^ Now, when it comes to chronic obstructive pulmonary disease and idiopathic pulmonary fibrosis, the constant Wnt/β-catenin activation is like pushing them into a state of aging and running out of stem cells.^30^^,^^31^ In the mammary gland, pregnancy induces robust activity of Lgr6^+^ basal and luminal stem cells leading to alveolar budding in response to hormonal stimuli. These same cells give rise to luminal breast cancer upon transformation.^5^

During kidney formation, Lgr6 marks progenitor cells in the metanephric mesenchyme, with expression kicking off pretty early on, around embryonic day 11.5.^2^^,^^14^^,^^25^ Lgr6-positive progenitor cells differentiate into renal tubular epithelial cells and podocytes to form nephrons.^2^^,^^23^ The Lgr6^+^ cells can generate nephron structures throughout life via mesenchymal-to-epithelial transition, which have persistent regenerative ability.^25^ Skeletal systems similarly depend on Lgr6^+^ progenitors for directly osteogenic differentiation of MSCs by stabilizing β-catenin to potentiate Wnt signaling,^42^^,^^43^^,^^48^^,^^50^ with fracture-triggered upregulation promoting callus mineralization.^47^^,^^49^ The list goes on, even when it comes to regrowing fingertips; Lgr6^+^ nail stem cells are absolutely key for getting things back to normal.^42^

### The differences in the role of Lgr6 as a stem cell marker (for example, skin and breast)

The identity of Lgr6 as a stem cell marker is strongly dependent on the tissue. In the hair follicle, Lgr6+ cells are located mainly in the bulge and function as a multipotent epidermal stem cell population that can give rise to several different cell types. Most recent lineage tracing experiments have demonstrated their multipotency and ability to generate all major skin lineages (HF, SG, and IFE).^4^^,^^23^^,^^132^ However, in the adult mouse mammary gland, Lgr6 marks only a sparse population of cells within the basal and luminal layers.^59^ Although these Lgr6^+^ cells exhibit stem/progenitor-like properties, their differentiation potential is far more limited compared with their counterparts in the skin. Lineage tracing experiments indicate that mammary Lgr6^+^ cells specifically produce luminal epithelial cells, which, when transformed by oncogenic mutations, can initiate luminal-type mammary tumors.^5^^,^^62^ Thus, in the mammary gland, Lgr6 appears to mark a committed luminal progenitor or a stem cell population with luminal bias, rather than multipotent stem cells as in the skin.

The crucial role of Lgr6^+^ stem cells in the epidermis hinges on the presence of nerves. Their presence, being close to nerve endings or Schwann cells, and their ability to help heal wounds are greatly affected when nerves are removed.^4^^,^^132^ Moreover, these stem cells' behavior is controlled by various signaling pathways within the surrounding environment, with the Wnt pathway standing out in its activation.^120^ The behavior of Lgr6^+^ stem cells in the mammary gland is notably shaped by hormonal factors, particularly those stemming from the ovaries and pregnancy.^59^

Lgr6^+^ and Lgr5^+^ cells are, for the most part, distinct populations that carve out their own territories.^5^^,^^23^^,^^116^^,^^124^ Lgr5^+^ cells are mainly found in the bulge, where they have a crucial role in HF regeneration. Conversely, Lgr6^+^ cells reside in the isthmus, SG, and IFE, where they contribute to long-term wound healing and HF neogenesis.^23^^,^^124^^,^^132^ They form separate stem cell pools.^116^ In addition, Lgr5 marks basal mammary stem cells with potency to regenerate the whole mammary gland, whereas Lgr6+ cells present in both basal and luminal layers are distinct from multipotent Lgr5+ basal mammary stem cells.^5^ In skin carcinogenesis, Lgr6 deficiency increases susceptibility to SCC. This is concomitant with the compensatory overexpression of Lgr5 and gain of Wnt signaling, thereby disturbing homeostasis and promoting tumor progression. In skin cancer, Lgr6 deficiency worsens the situation, leading to increased susceptibility to SCC accompanied by overcompensation by Lgr5 and gain of Wnt signaling, resulting in loss of homeostasis and tumor progression.^126^

### Abdomen

It has been reported that MaR1 activates Lgr6 signaling, thereby inhibiting smooth muscle cell activation and reducing abdominal aortic aneurysm formation in rodents.^16^ As a macrophage-derived peptide, MaR1 has the ability to activate Lgr6 receptor and further exert its immune clearance function, resolving acute inflammation and protecting organs.^11^^,^^133^ In both human and mouse phagocytes, MaR1 really ramps up the process of phagocytosis, boosts the intracellular digestion that occurs within these cells, and triggers the phosphorylation of a whole bunch of proteins, including ERK and cAMP response element binding protein. When Lgr6 is overexpressed, these effects of MaR1 are significantly amplified; however, they are diminished when Lgr6 gene silencing occurs in phagocytes.^16^ Thus, MaR1 acts as an endogenous Lgr6 activator, uncovering its role in enhancing MaR1's anti-inflammatory effects.

### Lgr6 and Wnt/β-catenin signaling pathway

In ovarian cancer tissue, Lgr6 enhances the Wnt/β-catenin pathway's function through its interaction with RSPOs, facilitating the advancement of high-grade serous ovarian carcinoma.^59^, ^60^, ^61^ This mechanism entails the activation of the Wnt/β-catenin signaling cascade and the enhancement of pluripotency-associated factors like SOX2 and OCT4.^63^ In non-small cell lung cancer, Lgr6 is specifically expressed in the tumor area and rises with disease advancement.^21^ Compared to Lgr6-cells, Lgr6+ cells at different stages of lung adenocarcinoma exhibit a greater ability for tumorigenicity and self-renewal, suggesting that the p38α and miR-17-92 pathways are imbalanced. This imbalance is marked by defects in the p38α signaling pathway, leading to the global dysregulation of Wnt signaling pathway components, promoting the inhibitory effect of Wnt inhibitors and the function of activators.^31^^,^^32^ As a promoter of Wnt/β-catenin signal transduction, Lgr6 enhances the response to Wnt ligands and amplifies receptor signaling, resulting in the specific survival of Lgr6^+^ cells during cancer progression.^18^^,^^35^ Additionally, Lgr6 participates in Wnt signaling within the nervous system, predominantly expressed in the central nervous system at 8.3 astrocytes.^72^ The binding of Lgr6 to its ligand, RSPO1, not only promotes the expression of Wnt signaling but is also strongly associated with Norrin proteins.^69^^,^^70^ In the realm of kidney development, Lgr6 acts as a key identifier for nephron progenitor cells, playing an essential role in shaping the renal tubular epithelium and podocytes.^23^ Additionally, Lgr6's expression, which triggers the Wnt signaling pathway, is instrumental in driving the growth and differentiation of both tubular epithelium and podocytes.^23^ Consequently, Lgr6 plays a key role in multiple cancers and kidney formation by regulating or enhancing Wnt signaling and its downstream targets. Simultaneously, the Wnt signaling pathway positively influences the expression and function of Lgr6, establishing a positive feedback loop.

MaR1, an anti-inflammatory lipid mediator, has been shown to have significant anti-inflammatory effects.^9^ Lgr6 serves as a crucial receptor for MaR1, playing a pivotal role in safeguarding against a range of illnesses. Earlier research has demonstrated that MaR1 effectively mitigates by curbing the generation of ROS, reducing inflammation, and preventing tissue fibrosis.^10^ The activation of Lgr6, one of the receptors of MaR1, can up-regulate the expression of cAMP. The increase in cAMP can further up-regulate the activity of SOD2 in a Lgr6 dependent manner. MaR1 could enhance SOD2 via the Lgr6-linked cAMP/SOD2 antioxidant mechanism by suppressing ROS, reducing hyperglycemia-related inflammation.^11^, ^12^, ^13^^,^^26^ This underscores the inseparable relationship between the effects of MaR1 and Lgr6.

RORα was proposed as a potential receptor of MaR1.^39^ However, experiments have confirmed that the activation of Lgr6 by MaR1 does not depend on RORα.^11^ Lgr6-mediated vascular smooth muscle cell inhibition and aortic aneurysm protection occur independently of the RORα pathway.^11^ This suggests that Lgr6 is the major direct receptor through which MaR1 exerts its anti-inflammatorypro-resolving functions, whereas RORα may be involved in signaling by other lipid mediators.

Lgr6 up-regulates SOD2 activity via the GPCR-cAMP second messenger system (non-Wnt pathway) that inhibits ROS production^13^^,^^22^^,^^26^; this pathway is in sharp contrast to the canonical Wnt/β-catenin pathway driven by Lgr4/5 (*e.g.*, intestinal stem cell maintenance),^14^ explaining its tissue-specific functional differences.

In summary, Lgr6 is the core functional receptor of MaR1, and its signaling mechanism (cAMP–SOD2–ROS axis) is independent from LGR4/5 and RORα, which provides a molecular basis to target the Lgr6-MaR1 axis for the treatment of metabolic diseases, such as diabetic nephropathy and inflammation.^10^^,^^16^^,^^22^

### Interaction of Lgr6 with other receptors in MaR1 signaling

MaR1 has been shown to be the endogenous ligand for Lgr6 (but not Lgr4 or Lgr5), operating through a unique signaling pathway. MaR1-induced activation of Lgr6 triggers Gαs-cAMP signalling to enhance macrophage phagocytosis while resolving inflammation.^18^ In contrast, knockdown of Lgr6, but not Lgr4/5, significantly attenuates MaR1-induced cAMP elevation, ERK phosphorylation and efferocytosis,^18^^,^^37^ underscoring Lgr6's exclusive role in MaR1 signaling. While RORα was initially proposed as a potential MaR1 receptor,^27^ subsequent studies revealed that Lgr6 activation by MaR1 occurs independently of RORα, as demonstrated by Lgr6-mediated vascular smooth muscle cell regulation and aortic aneurysm protection.^18^ Therefore, Lgr6 is now believed to be the principal receptor for MaR1 and its anti-inflammatory and pro-resolving effects, while RORα may potentially mediate other lipid mediator signalling pathways. Mechanistically, Lgr6 activates SOD2 via a GPCR-cAMP secondary messenger system (independent of Wnt) to suppress ROS production.^19^^,^^21^^,^^22^ This contrasts sharply with the canonical Wnt/β-catenin signaling associated with Lgr4/5, which governs processes like intestinal stem cell maintenance.^14^^,^^38^ In essence, Lgr6 serves as the linchpin receptor for MaR1, and its unique “cAMP–SOD2–ROS” axis, independent of LGR4/5 and RORα, lays the groundwork for therapeutic targeting of the Lgr6-MaR1 pathway in metabolic and inflammatory disorders, including diabetic nephropathy.^19^^,^^20^^,^^37^

### Outlook

Lgr6-centered approaches hold promise in the treatment of non-small cell lung cancer and ovarian cancers through regulation of the Wnt/β-catenin pathway. In non-small cell lung cancer, Lgr6 is overexpressed in tumor tissue, driving Wnt/β-catenin-mediated oncogenicity and stemness.^18^^,^^21^ Preliminary research involving the suppression of Lgr6 (such as through siRNA) has demonstrated its potential in curbing tumor proliferation and resistance to chemotherapy.^62^ However, several challenges remain for cancer therapy, including the homology between Lgr6 and related Lgr4/Lgr5 and concomitant toxicity^14^; and the possibility of Lgr5 overcompensation upon Lgr6 inhibition might even lead to therapy resistance.^127^

While Lgr6 typically promotes tumor growth in cancers, it serves a beneficial function in certain regenerative medicine applications. In wound repair, Lgr6-positive epidermal stem cells stimulate tissue regeneration and hair follicle formation, suggesting potential therapeutic use of Lgr6^+^ cell treatments or activators for chronic wounds. However, a key limitation lies in the nerve-dependent activation of Lgr6 in tissues lacking nerve supply.^116^ Conversely, siRNA- or CRISPR-mediated inhibition of Lgr6 accelerates fracture healing via promoting osteoblast development, but appropriate timing and localization are required to preserve normal bone turnover.^45^^,^^47^ These tissue-specific actions highlight the importance of developing delivery methods that consider the diverse functions of Lgr6 in distinct biological processes.

By 2025, researchers have not launched any clinical trials focusing on Lgr6,^14^ mainly because we are still in the dark about its ligand-receptor interactions and have valid safety worries.^18^^,^^127^ Moving forward, the next wave of studies must lean on *in vivo* models that are a closer match to human biology. This approach will help us unpack the intricacies and evaluate safety, ultimately confirming whether Lgr6 is a viable therapeutic target.

### Lgr6 as a diagnostic and prognostic biomarker

Lgr6 has great potential as a biomarker for the diagnosis and prognosis of several cancers. In colorectal cancer, high levels of Lgr6 mRNA in lymph nodes are associated with shorter disease-free survival and represent an independent prognostic factor. Thus, Lgr6 is a good candidate to help stratify the risk of postoperative recurrence together with CEA and CXCL16.^81^ Furthermore, Lgr6 is markedly overexpressed in esophageal SCC tumor tissue compared with healthy esophageal tissue, and its expression escalates with advancing TNM stage and poorer differentiation, bolstering its potential as a diagnostic marker.^82^ In gastric cancer, Lgr6 fuels disease progression through the PI3K/AKT/mTOR signaling pathway and correlates with aggressive traits like invasion and metastasis.^79^^,^^84^ When it comes to ovarian cancer, Lgr6 plays a role in chemoresistance and dictates treatment response.^59^^,^^60^ Lastly, in non-small cell lung cancer, Lgr6 pops up exclusively in tumor tissue, absent from adjacent normal lung, and its expression climbs as the disease progresses.^21^ The AUC of the receiver operating characteristic (ROC) curve for diagnosing esophageal SCC was not explicitly stated. ^82^ However, findings on lymph node prognostication in colorectal cancer demonstrate good discriminatory power,^81^ but further testing in larger cohorts will be required for translation to the clinic.

### Research limitations

Despite a thorough examination of Lgr6's contribution to multi-tissue homeostasis and healing processes, the prevailing models present notable gaps. For one, when examining Lgr6 knockout mice, it is essential to recognize that other Lgr family members, like Lgr5, can skew the outcomes via an overcompensation mechanism.^126^^,^^130^ This compensatory effect is particularly pronounced in skin cancer, where overexpression of Lgr5 induced by Lgr6 knockout may activate canonical Wnt signaling ectopically, confounding the interpretation of Lgr6-specific effects.^126^ Secondly, tissue heterogeneity complicates the interpretation of Lgr6 function. On one hand, Lgr6 knockdown promotes osteogenic differentiation of BMSCs *in vitro*,^45^ yet in a mouse fracture model, Lgr6 deficiency can hinder bone repair.^47^ These conflicting results underscore the intrinsic limitation of conventional bulk analysis to delineate cell subgroup-specific mechanisms. Future studies should combine cutting-edge single-cell omics and gene-edited organoids to dissect the cell type-specific Lgr6 pathway without interference from compensatory mechanisms for clinical translation.

## Conclusion

Lgr6 exhibits dual value as a disease diagnostic marker and therapeutic target. In cardiovascular and cancer diseases, changes in their expression levels can serve as biomarkers for evaluating disease progression, prognosis, and treatment response. As a potential therapeutic target, research on small-molecule drugs or antibodies targeting LGR6 aims to regulate related pathological processes. Its role in adult stem cells also provides a new perspective for tissue regeneration and the treatment of degenerative diseases. However, there are still key bottlenecks in Lgr6 research. The spectrum of its ligand recognition (such as the competitive binding of RSPOs and MaR1) and the spatiotemporal dynamic regulation of the Wnt/BMP/PI3K pathway after activation have not been clarified. The “double-edged sword” effect of Lgr6^+^ cells in cancer, which promotes breast cancer occurrence but inhibits colon cancer metastasis, and the mechanism of loss of function in aplastic anemia urgently need to be elucidated, requiring the establishment of tissue-specific gene editing models and aging-regeneration dynamic evaluation systems. There is a lack of spatiotemporally precise targeted intervention strategies, inconsistencies in clinical marker detection standards, and limitations in the transplantation efficiency of Lgr6^+^ cells. Therefore, a deep analysis of the Lgr6 signaling network is crucial for the development of novel targeted drugs and the regulation of disease processes, but systematic breakthroughs in the aforementioned mechanisms, pathology, and translational challenges are needed.

## CRediT authorship contribution statement

**Han Li:** Writing – original draft, Investigation, Resources. **Xiaoqi Guan:** Resources, Data curation. **Yu Wang:** Supervision, Conceptualization. **Haidong Guo:** Supervision, Writing – review & editing, Conceptualization.

## Data availability

Any data pertaining to our research can be obtained upon request to the corresponding author.

## Funding

This work was supported by grants from the 10.13039/501100001809National Natural Science Foundation of China (No. 82174120), Traditional Chinese Medicine Research Project of Shanghai Municipal Health Commission of China (No. 2024PT005), and Program of Shanghai Academic Research Leader (China) (No. 22XD1423400).

## Conflict of interests

The authors declared no conflict of interests.
